# Thermal Stress and Temperature Analysis of Integrated Protection-Thermal Control Multilayer Films Under Laser Irradiation in Alternating High and Low Temperatures

**DOI:** 10.3390/ma19183817

**Published:** 2026-09-08

**Authors:** Chao Zhou, Rui Zhu, Shengzhu Cao, Jun Yang, Binhua Gui

**Affiliations:** 1 School of Electronic and Information Engineering, Lanzhou Jiaotong University, Lanzhou 730070, China; zhurui_pink123@163.com (R.Z.); mhxyforever@126.com (J.Y.); guibinhua@163.com (B.G.); 2 Science and Technology on Vacuum Technology and Physics Laboratory, Lanzhou Institute of Physics, Lanzhou 730000, China; caoshengzhu@126.com

**Keywords:** laser protection, thermal control function, multilayer films, space environment, thermal stress

## Abstract

**Highlights:**

- Temperature and stress evolution in multilayer thin films was conducted under simulated space environments and laser irradiation;
- Address the technical bottleneck wherein the stress-state evolution among multilayer films and the macroscopic temperature evolution are difficult to obtain through experimental measurement methods;
- COMSOL Multiphysics^®^ software (version 6.3) was employed as a simulation and computational tool to analyze the stress states between different film layers, thereby guiding the design of multilayer thin-film systems.

**Abstract:**

With the rapid advancement of space-based laser weapon technologies, on-orbit safety of spacecraft such as satellites is confronted with severe laser threats. To meet the demands for film system optimization and reliability improvement of thin films integrating space laser protection and thermal control functions, this study takes the Graphene/Ag/Al_2_O_3_/SiO_2_/ITO multilayer thin film structure as the research object. Combined with the space alternating high-low temperature environment and the action of ultra-high-density transient directional heat flux, systematic simulation research on the evolution laws of temperature and stress fields inside the multilayer thin films under laser irradiation in alternating space high-low temperature environments is carried out via COMSOL Multiphysics, and the influencing mechanisms of ambient temperature and laser operating parameters on thermal stress and temperature distribution are revealed. The simulation results demonstrate that the thin film structure reaches thermal equilibrium within several seconds under a given transient directional heat flux. As laser power rises, the peak temperature of each layer increases nonlinearly and the time required to reach thermal equilibrium shortens. The laser heat flux density acts as the dominant factor governing the temperature and thermal stress distribution. Under alternating space high-low temperature conditions, the thermal stress of the thin film varies approximately linearly with temperature while the overall stress magnitude remains low, and thermal stress is mainly concentrated in the Al_2_O_3_ layers. Laser loading exerts a remarkable impact on film thermal stress: the amplitude of thermal stress in all film layers rises synchronously with increasing laser power, and interlayer temperature gradients as well as stress concentration are further intensified. The stress growth of Ag and Al_2_O_3_ layers is the most significant, which can be attributed to the synergistic effect of interlayer thermal expansion coefficient mismatch and temperature gradients. The alternating high-low temperature and laser irradiation experiments indicate that the maximum temperature and maximum stress borne by the muti-layer film under alternating temperatures ranging from −150 °C to 150 °C and laser irradiation of 200 W/cm^2^ will not lead to macroscopic failure behaviors and degradation of thermal control performance.

## 1. Introduction

The rapid evolution of novel threats such as space-based laser weapons poses severe challenges to the on-orbit protection capacity of spacecraft including satellites, and has become a critical technical field requiring urgent breakthroughs in the aerospace industry [1,2,3,4,5]. As an essential component of spacecraft exterior surfaces, thermal protection systems undertake dual missions: first, they shield core components by reflecting and absorbing laser energy; second, they stabilize the internal temperature field via thermal regulation mechanisms [6,7,8]. When laser irradiation acts on thin-film structures, remarkable temperature gradients form inside the films. Discrepancies in the thermal expansion coefficients of different film layers lead to inconsistent interlayer deformation, which triggers the generation and accumulation of thermal stress. This further causes film cracking, interfacial debonding and even substrate deformation, exerting adverse impacts on key optical and electrical properties of the thin films [9,10].

To address the above issues, the integrated functional design concept has been proposed in the field of satellite laser protection, which integrates laser protection and thermal control functions into a single thin-film structure. Such integrated multilayer thin films adopt highly reflective layers to efficiently dissipate incident laser energy on the surface by virtue of their high reflectivity at specific wavelengths, thereby restraining heat transfer into the substrate from the source and effectively lowering the peak temperature and internal temperature gradients of the films [11,12,13]. By blocking deep heat deposition, the amplitude of thermal stress induced by mismatched thermal expansion coefficients between layers is significantly reduced, which suppresses thermal damages such as interfacial debonding and film cracking caused by stress concentration, and realizes coordinated protection for thin-film structures and their substrates [14,15]. Laser irradiation essentially corresponds to the formation of spatially inhomogeneous temperature fields under ultrahigh-density transient directional heat flux. Such temperature fields give rise to divergent thermal expansion behaviors of materials, induce the generation and concentration of thermal stress, and may further initiate and propagate cracks [16]. Therefore, although high-reflection designs substantially inhibit heat deposition, the inhomogeneity of temperature fields and the accompanying thermal stress distribution remain core factors determining the long-term reliability of thin films. Systematically revealing the corresponding laws carries important theoretical exploration value and engineering application significance for optimizing film systems and improving laser damage resistance thresholds [17].

In recent years, research on laser protection has mainly focused on developing high-reflectivity and high-absorptivity materials, represented by metallic coatings and carbide coatings [18]. In terms of thermal control, multi-layer insulation materials and aerogels have been widely applied in spacecraft thermal control systems [6]. Nevertheless, integrated thin films combining laser protection and thermal control need to tackle the technology of rapid heat dissipation under high-density transient heat flux. For instance, ultrahigh reflectivity at specific laser wavelengths (e.g., 1064 nm) is required, while critical thermal control performances including surface absorptivity and emissivity must be simultaneously satisfied. Specifically, targeted emissivity or absorptivity is demanded across the full thermal infrared band (8~14 μm in general) or solar spectrum band (0.3~2.5 μm): high emissivity for heat dissipation and low emissivity for thermal preservation, so as to meet the normal thermal control demands of on-orbit satellite components. Accordingly, the film system designs for laser protection often conflict with those required to achieve ideal thermal control performance, and research on integrating laser protection and thermal control functions into one single multilayer thin-film structure remains limited. High-energy laser reflective protection mainly relies on surface coating technology and structural protection is realized by depositing laser-resistant thin films on substrates. Institutions including Lanzhou Institute of Physics [19], Changchun Institute of Optics, Fine Mechanics and Physics, Chinese Academy of Sciences [20], and General Department of China Academy of Space Technology [6] have carried out research on high-energy laser protection technologies based on high-reflectivity materials targeting satellite thermal control multilayers and large-area exterior thermal control materials, and relevant technologies have been implemented on orbit. However, systematic research on the thermal stress behaviors of the above multilayer thin films under laser irradiation is still lacking, and the underlying mechanisms have not been fully clarified.

Aiming at the demands for film system optimization and reliability improvement of thin films integrating laser protection and thermal control functions, this paper takes the Graphene/Ag/Al_2_O_3_/SiO_2_/ITO multilayer thin film as the research object. The structural design of this multilayer film realizes coordinated matching of photothermal performances among functional layers, delivering high laser reflectivity and high infrared reflectivity, together with low solar spectral absorptivity and high infrared emissivity. Existing studies on integrated laser-protection and thermal-control thin films mainly focus on boosting the photothermal performance indicators of thin films, establishing thermal stress analysis theories for multilayers, and investigating the space environmental stability of multilayer coatings. Differences in thermal expansion coefficients and lattice constants between dissimilar materials lead to considerable internal stress within film systems during temperature variations. Especially for high-energy laser protection applications, irradiation with ultrahigh heat flux of 100 W/cm^2^ can raise the overall temperature of the film system by hundreds or even thousands of degrees within several seconds. Such large temperature differences inevitably generate severe thermal stress, which triggers film failures such as delamination and peeling. By establishing a thermo-mechanical multi-physics coupling analysis model for multilayer thin-film structures and investigating the distribution and evolution of temperature and thermal stress within film systems subjected to laser irradiation and alternating space high-low temperature environments, this paper proposes optimization and improvement strategies for integrated laser-protection and thermal-control thin films. Thermal stress can be reduced via optimizing the thickness of each functional layer and introducing transition layers, so as to obtain stress-optimized film schemes. Iterative optimization is further conducted together with optical performance optimization models to form flexible thin-film design schemes integrating high-energy laser protection and thermal control functions. This work intends to provide scientific basis and optimization guidance for the protection design of spacecraft.

## 2. Materials and Methods

### 2.1. Multilayer Thin Film Materials and Structures

**Figure 1:**
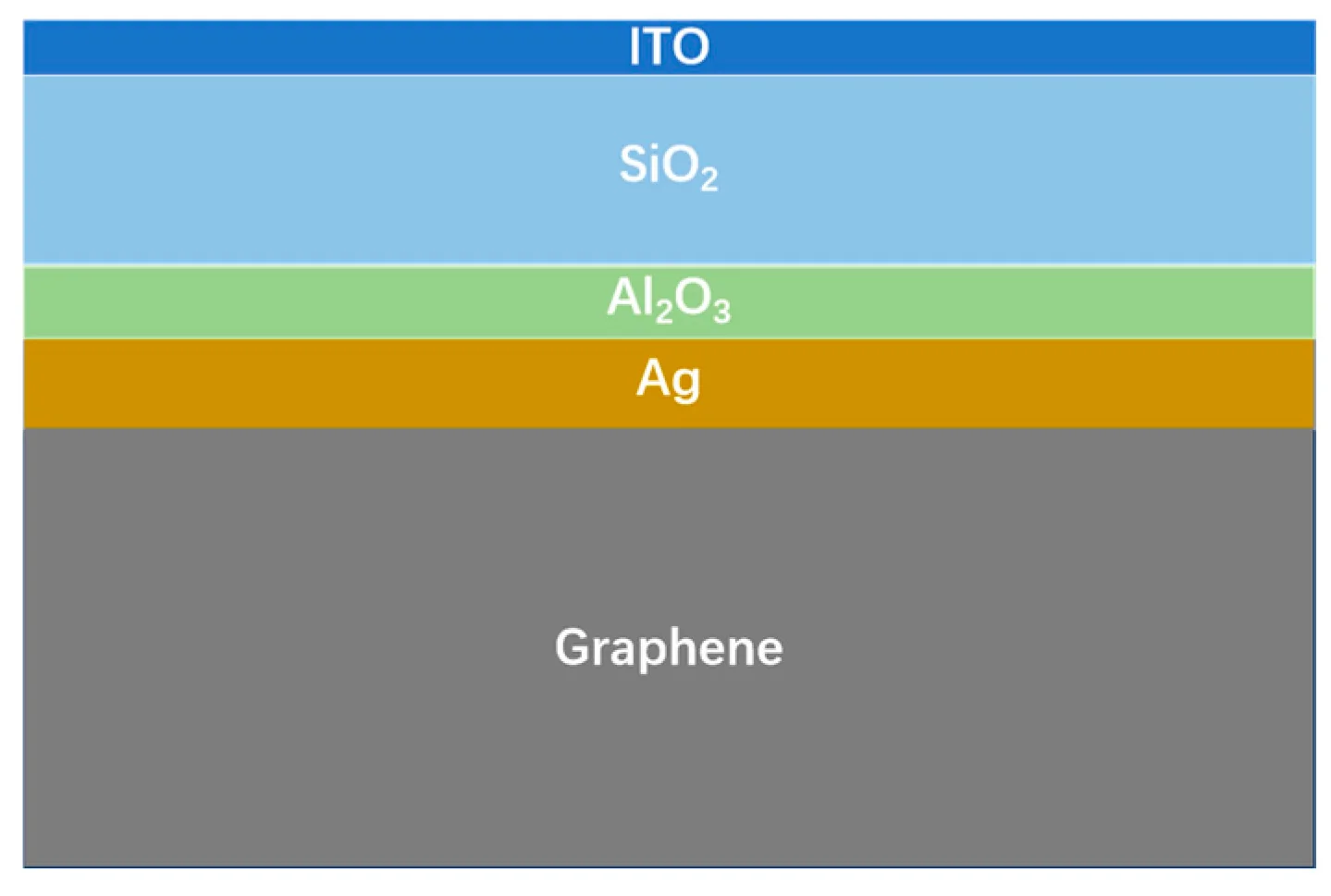
The multilayer film s materials and structures.

The multilayer thin film materials and structures integrating laser protection and thermal control functions are composed of Graphene/Ag/Al_2_O_3_/SiO_2_/ITO, as shown in Figure 1. The thickness and material system of each functional layer are listed in Table 1. Each functional film layer is sequentially deposited by Physical Vapor Deposition (PVD), and the corresponding preparation methods are given in Table 2. Among them, the Ag and ITO layers are prepared by DC magnetron sputtering, while the Al_2_O_3_ and SiO_2_ layers are deposited by IBO (Ion Beam Oxidation) combined with pulsed reactive magnetron sputtering. The deposition parameters are summarized in Table 3.

Graphene serves as the substrate material with high thermal conductivity. Ag acts as a high-reflectivity metallic layer to reflect laser energy and reduce direct laser interaction with the film [20]. The Al_2_O_3_ anti-reflective film functions as a transition layer with high thermal conductivity, offering protection by reducing energy absorption coupling [21]. The SiO_2_ film serves as a heat dissipation layer, which not only exhibits exceptionally excellent optical properties within the optical transmission band, but also possesses outstanding thermal stability, chemical stability, thermal shock resistance, and electrical insulation properties. SiO_2_ thin films are the best choice for low-refractive-index optical coating materials from the ultraviolet to the near-infrared range [22]. The transparent conductive oxide ITO is currently the most outstanding transparent conductive material in terms of optoelectronic performance. Owing to its combination of high visible-light transmittance and low resistivity, as well as low infrared emissivity and high reflectivity, it is an excellent material for fabricating the multilayer thin film structure that integrates laser protection and thermal control functions [23].

### 2.2. Theory of Thermal Stress Analysis Using Heat Conduction Equation

Under laser irradiation and the high-low temperature environment in space, a large number of free-moving electrons inside the thin film structure move under the action of the laser electric field. The motion and oscillation of these electrons are converted into thermal energy, causing the temperature of the protective film to rise. The temperature distribution can be described by the heat conduction equation [24]. The temperature variation of the protective film over time is typically described by Fourier’s law:

(1) $$\rho C_{P}\frac{\partial T}{\partial t}+\nabla (-k\nabla T)=Q$$

where, *ρ*—density; *C_P_*—heat capacity, i.e., the amount of heat absorbed by the protective film per 1 K temperature rise when its temperature increases due to absorbed heat; *k*—thermal conductivity, an intrinsic physical property of the protective film material, which varies with time, temperature, and other parameters; *Q*—laser energy source term, i.e., the heat flux density, representing the heat absorbed by the protective film. It can be expressed in terms of the laser power density *I* and the absorption coefficient *k* (*x*—position coordinate).

(2) $$Q(x,t)=I\cdot k\cdot e^{-kx}$$

Transient thermal analysis was conducted based on the heat conduction equation to obtain the temperature field distribution of the protective film structure. The calculated temperature field was then applied as the thermal load in the subsequent stress analysis to determine the stress distribution within the protective film structure. According to Hooke’s law, the stress and strain in the *i*-layer film satisfy the following relationship:

(3) $$\sigma_{i}=E_{i}(\varepsilon -\alpha_{i}\Delta T)$$

where, $\sigma_{i}$ is thermal stress, $E_{i}$ is Young’s Modulus, ε is thermal strain, $\alpha_{i}$ is coefficient of thermal expansion, Δ*T* is Temperature change.

Under the same temperature variation Δ*T*, there exists a difference in the theoretical free thermal strain of each film layer, resulting in thermal strain mismatch. The larger the difference in thermal expansion coefficients between multilayer materials and the greater the temperature variation Δ*T*, the greater the thermal strain mismatch, the more prominent the constrained strain between layers, and the higher the magnitude of thermal stress initiated at the interfaces. According to the approximate solution proposed by Townsend et al. [25], thermal stress is proportional to the difference in thermal expansion coefficients between adjacent layers.

Thermal stress generated in multilayer thin-film structures under alternating high and low space temperatures and laser thermal loading conditions essentially originates from the coupling effect of mismatched interlayer thermophysical parameters and interfacial displacement constraint effects. Interfaces with greater thermal expansion coefficient mismatch exhibit higher stress concentration coefficients during temperature variations. As the number of film layers increases, peak stress occurs at the interface between adjacent layers with the largest differences in elastic modulus and thermal expansion coefficient. In a multilayer system, each newly formed interface introduces additional mismatched strain, resulting in progressive stress accumulation across successive layers. Once local thermal stress exceeds the fracture strength of the film material or the interfacial bonding strength, film cracking or interfacial delamination will occur, degrading the reliability of the multilayer structure.

### 2.3. Multi-Field Coupling Simulation Analysis

COMSOL Multiphysics is a large-scale numerical simulation software based on the finite element method. It enables direct, bidirectional and real-time coupling of an arbitrary number of physical fields. Therefore, this software is adopted in this paper to carry out coupled heat conduction and thermal stress analysis, so as to investigate the distribution characteristics of temperature field and stress field of multilayer thin films under laser irradiation and alternating high-low temperature space environments. All simulations were performed using the COMSOL Multiphysics^®^ software (version 6.3) [26]. The Heat Transfer in Solids and Solid Mechanics modules were employed for the coupled thermal-stress analysis.

(1) A geometric model of Graphene/Ag/Al_2_O_3_/SiO_2_/ITO was established according to the structure of the multilayer thin film. The thickness of each layer is specified as follows: Graphene (20 μm), Ag (150 nm), Al_2_O_3_ (100 nm), SiO_2_ (2 μm), and ITO (50 nm). The total overall thickness is 22.3 μm.

(2) Material parameters: Corresponding parameters including thermal conductivity, specific heat capacity, elastic modulus and thermal expansion coefficient were set according to the thermophysical properties of each thin-film layer, as listed in Table 4.

(3) The physics interfaces of Solid Mechanics and Solid Heat Transfer were configured. An ultra-high-density transient directional heat flux was applied on the laser irradiation surface to simulate laser illumination. Alternating high and low temperature space thermal conditions were imposed on the entire multilayer structure to reproduce the thermal environment of satellites in orbit, with temperature T varying from −150 °C to +150 °C. The laser power density P was set to range from 20 to 200 W/cm^2^, and the laser action area corresponds to a circle with a diameter of 1.2 cm. The surface of the model is subjected to radiative heat loss with an infrared emissivity of *ε* = 0.53. The side surfaces are treated as adiabatic because their area is negligibly small compared with the surface.

(4) A mesh convergence study was performed to ensure numerical accuracy. The model employs COMSOL’s predefined ‘ultra-fine’ mesh. The peak temperature at the laser spot center and the maximum thermal stress at the Ag/Al_2_O_3_ interface were monitored as convergence criteria. The differences between the ‘ultra-fine’ mesh and a finer custom mesh were less than 2% for temperature and less than 3% for stress, confirming mesh independence.

### 2.4. Experimental Verification and Analysis

High-low temperature cycling tests were carried out on the Graphene/Ag/Al_2_O_3_/SiO_2_/ITO multilayer thin-film structure. The test temperature range was set from −150 °C to 150 °C to simulate the extreme alternating hot and cold working conditions of satellites in orbit. A vacuum-environment laser-induced damage threshold test system for thin-film materials was adopted to evaluate the protective performance of the multilayer film under different ambient temperatures (−150 °C, 25 °C, 150 °C) and various laser loading powers. The test conditions for laser protection performance are listed in Table 5. The surface morphologies of the film before and after the test are analyzed using Zeiss Sigma 500 scanning electron microscope (SEM; Carl Zeiss Microscopy GmbH, Jena, Germany).

## 3. Results and Discussion

### 3.1. Simulation Analysis of Temperature Field Distribution

**Figure 2:**
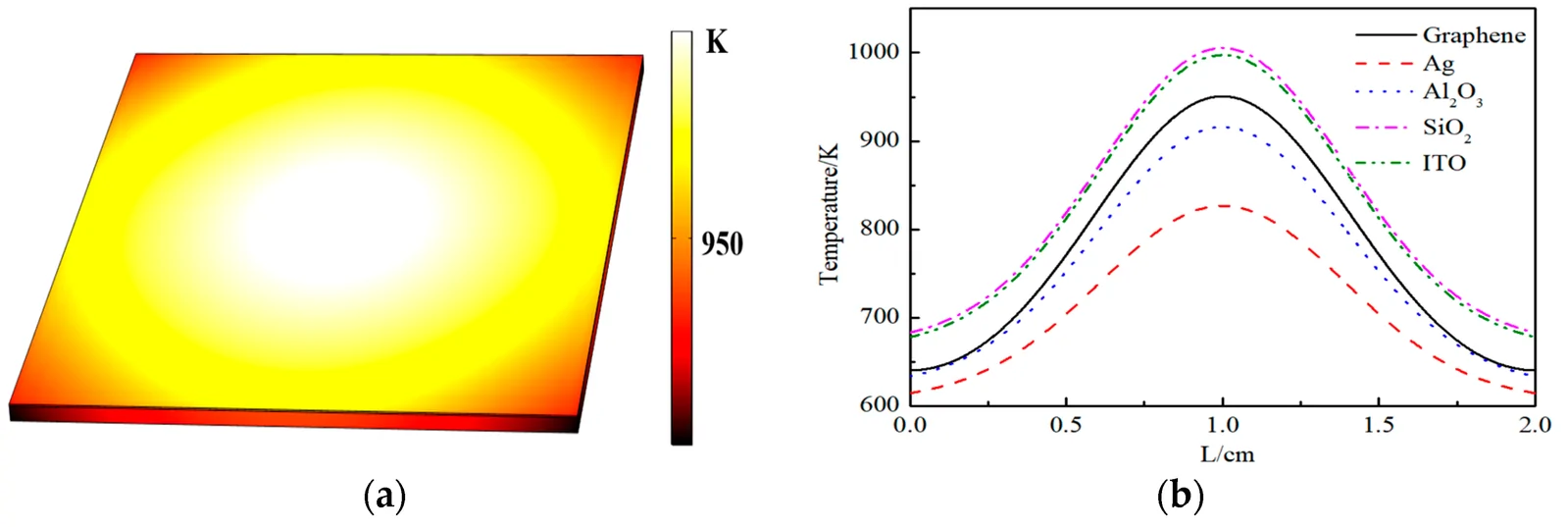
Temperature field distribution of the laser protective film structure at T = 150 °C, P = 100 W/cm^2^, t = 30 s. (**a**) Surface temperature distribution of the multilayer film. (**b**) Temperature distribution along the in-plane direction (L = 1.0 cm is the center point of laser irradiation).

Figure 2 presents the temperature field distribution of the laser protective film structure after 30 s under laser power density of 100 W/cm^2^ in temperature of 150 °C. It can be observed that under the effect of directional heat flux, the in-plane temperature distribution of the multilayer thin film matches the energy distribution of the Gaussian beam spot, with the maximum temperature occurring at the center of the Gaussian spot.

This non-uniform Gaussian temperature distribution not only alters the overall thermal response characteristics of the film and induces inhomogeneous in-plane thermal expansion deformation of the thin film, but also further intensifies the local thermal mismatch between adjacent layers of the multilayer structure, generating a differentiated stress distribution featuring high stress at central interfaces and low stress near edges. It serves as a critical inducement for local thermal damage and interfacial failure of thin films. Accordingly, during film system design, it is advisable to broaden the effective spot area or adopt thermal diffusion layers to suppress the temperature difference between the spot center and periphery, so as to minimize the risk of local thermal damage.

**Figure 3:**
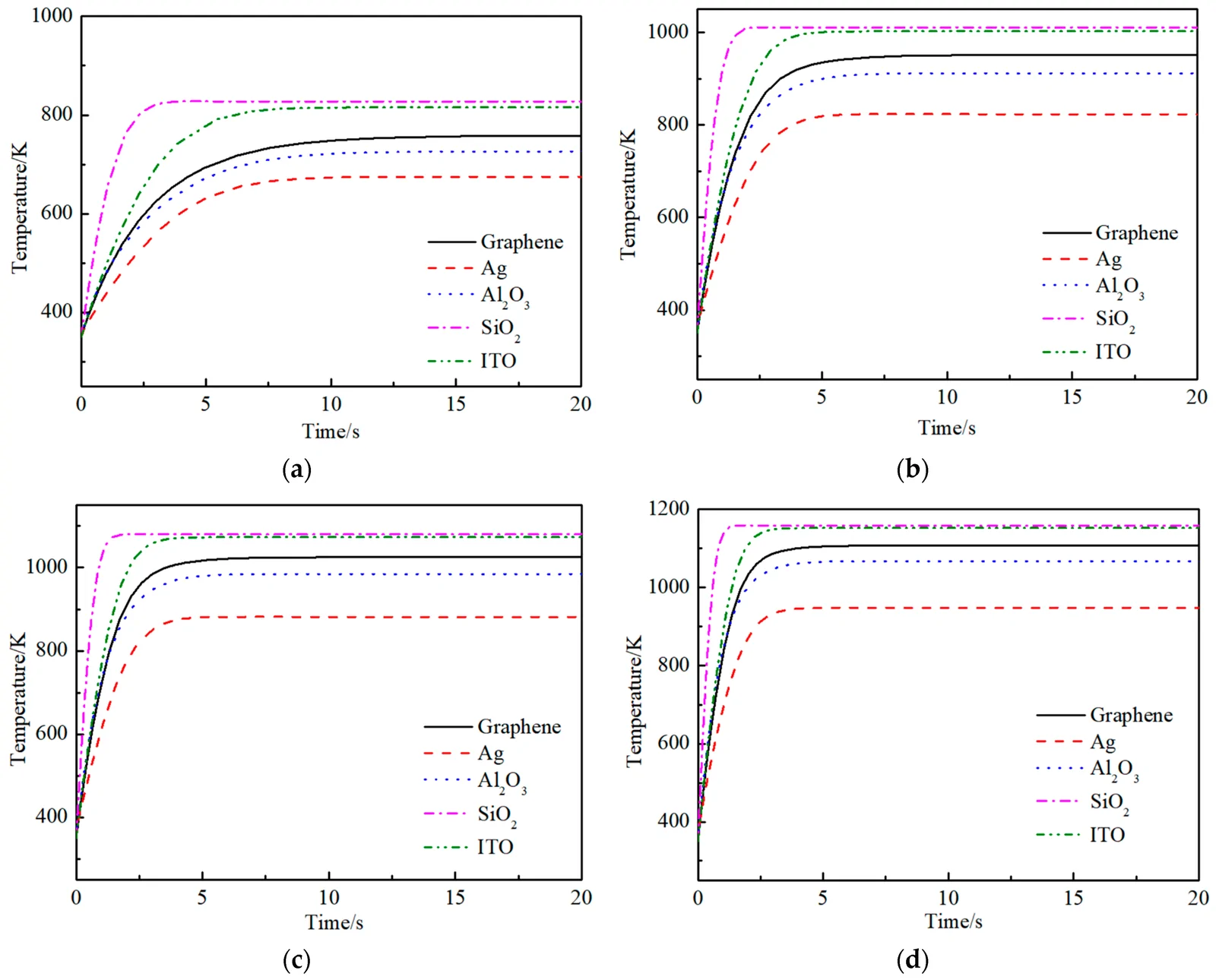
Variation of film temperature with laser irradiation time under different laser powers at T = 300 K, (**a**) P = 50 W/cm^2^, (**b**) P = 100 W/cm^2^, (**c**) P = 150 W/cm^2^, (**d**) P = 200 W/cm^2^.

Figure 3 illustrates the temperature evolution of each film layer with laser irradiation time under different laser power densities at an ambient temperature of 300 K. The temperature reaches a steady-state value within a few seconds after laser irradiation, and the peak temperature of each layer rises nonlinearly with increasing laser power. The Ag layer, serving as a thermal buffer layer, shows a lower temperature rise rate compared to its adjacent upper and lower layers. Under high-power conditions, the time required for each layer to reach thermal equilibrium is shortened, indicating that high-power laser induces more rapid heat accumulation inside the films, resulting in a steeper temperature gradient.

Differences in the internal temperature field distribution of the film are mainly governed by the thermal conductivity of constituent materials. It can be seen that the SiO_2_ and ITO layers have relatively low thermal conductivities and experience the most dramatic temperature rise. At a laser power density of 100 W/cm^2^ after irradiation of 30 s, their temperature reaches approximately 1000 K. In contrast, the Ag and Al_2_O_3_ layers, owing to their higher thermal conductivities, experience temperature increases to 823 K and 911 K, respectively. This indicates that the Ag and Al_2_O_3_ layers play a critical role in conducting and dissipating the incident heat flux, while the SiO_2_ layer, with its thermal insulation effect, effectively protects the interior of the laser protection film.

At a laser power of 200 W/cm^2^, the maximum temperature reaches approximately 1150 K. Since the lowest melting point among the multilayer film constituents is that of Ag, at approximately 1235 K, the peak temperature attained remains below the melting point of any material. Consequently, the multilayer film structure does not exhibit macroscopic ablation behavior induced by high temperature. Thermal stress induced by thermal expansion coefficient mismatch and temperature gradients is likely the primary cause of structural failure for the thin film. Accordingly, further analysis on thermal stress of the film structure is carried out.

The thickness of the integrated multilayer thin film is much smaller than the thermal diffusion length of the film and the substrate. Therefore, only heat conduction within the material is considered, i.e., all boundaries of the material model are assumed to be adiabatic, and the temperature is a function of time [26]. The research results indicate that laser power is the key parameter determining the temperature field distribution of the film. As laser power increases, the non-linear growth of the temperature gradient will significantly elevate the risk of thermal damage. Therefore, targeted optimization measures should be adopted in the film system design, such as increasing the Ag layer thickness or introducing a gradient transition layer, to minimize the degree of thermal stress concentration.

### 3.2. Simulation Analysis of Thermal Stress Distribution

#### 3.2.1. Stress Distribution Under High and Low Temperature Environment Without Laser Loading

The non-uniform temperature distribution within the film causes different degrees of thermal expansion and contraction across various regions of the film, thereby inducing a non-uniform distribution of thermal stress. To analyze the effect of laser irradiation on thermal stress in the film, the thermal stress of the film without laser loading under space high and low temperature environment (−150 °C~+150 °C) was first investigated. The “Thermal Expansion” node under the “Linear Elastic Material” option in the “Solid Mechanics” physics interface was employed for simulation.

**Figure 4:**
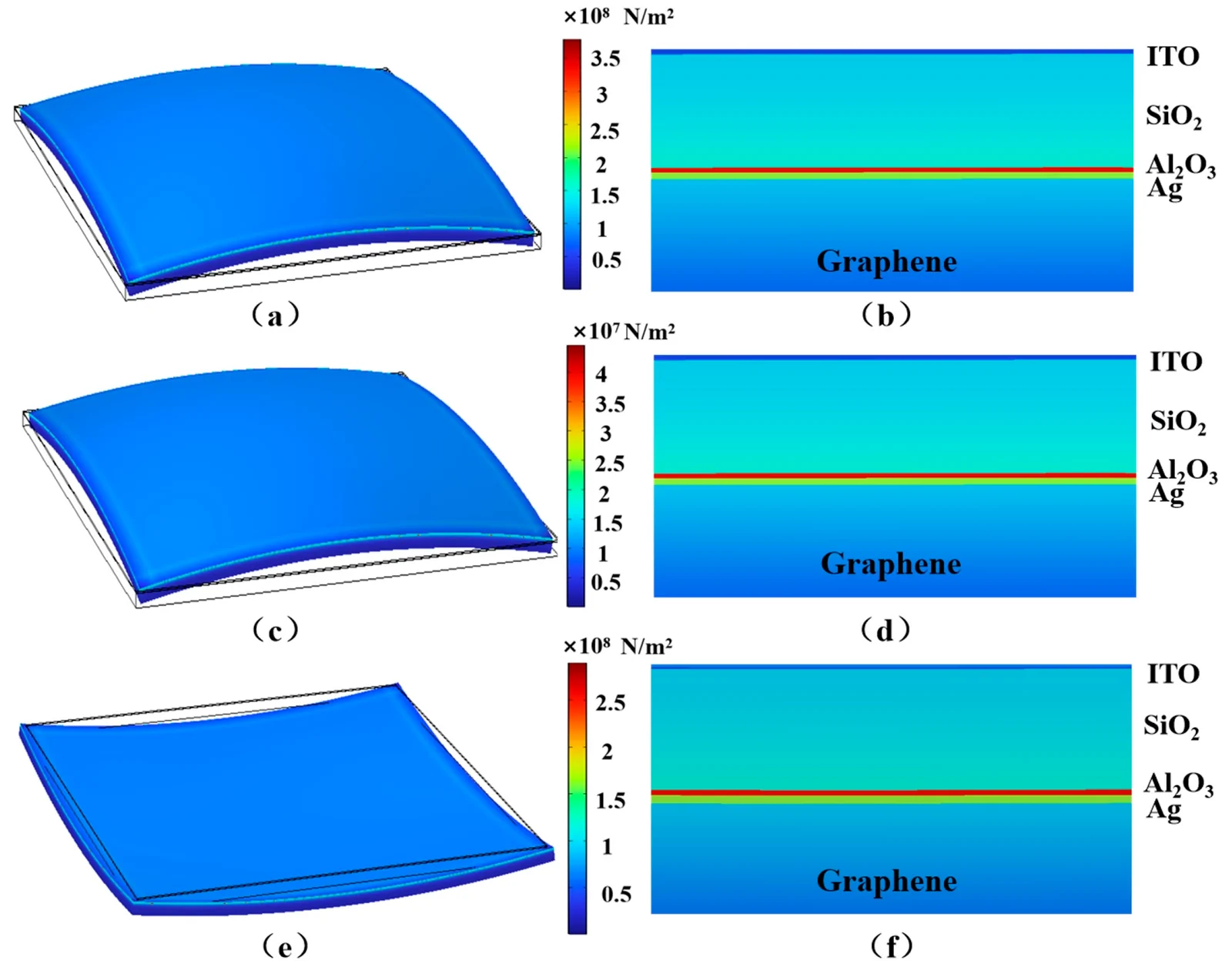
Thermal stress distribution of the multilayer film under temperature variation from −150 °C to 150 °C. (**a**) T = −150 °C, overall structural stress distribution. (**b**) T = −150 °C, stress distribution along the cross-section. (**c**) T = 0 °C, overall structural stress distribution. (**d**) T = 0 °C, stress distribution along the cross-section. (**e**) T = 150 °C, overall structural stress distribution. (**f**) T = 150 °C, stress distribution along the cross-section.

Under large temperature differences, thermal stress arises within the film due to the mismatch in thermodynamic properties between the multilayer thin films. The von Mises stress is used for relative comparison among layers. Figure 4 presents the thermal stress distribution of the multilayer film under temperature variations from −150 °C to 150 °C. The results indicate that within this temperature range, the multilayer film exhibits significant alternating tensile-compressive thermal stresses owing to differences in the coefficients of thermal expansion (CTE) among the layers. Under extreme temperature conditions of −150 °C and 150 °C, the interlayer stresses (on the order of several hundred MPa) are an order of magnitude higher than those at room temperature (on the order of tens of MPa). Along the cross-sectional direction, apparent stress discontinuities occur within the film. The Al_2_O_3_ layer exhibits relatively high stress, whereas the surface ITO layer and the Graphene substrate show lower stress levels due to sufficient strain accommodation space. This non-uniform stress distribution primarily arises from the significant differences in thermal expansion coefficients and elastic moduli among the constituent materials, resulting from incompatible constrained deformation during temperature variations. Under long-term service conditions, stress concentration regions are prone to induce interfacial delamination or microcrack initiation, thereby compromising the overall reliability of the protective film. The future work will focus on a comprehensive stress state analysis, including tensile/compressive principal stresses and interfacial shear stress, to better understand the specific failure mechanisms such as brittle cracking and interface delamination.

**Figure 5:**
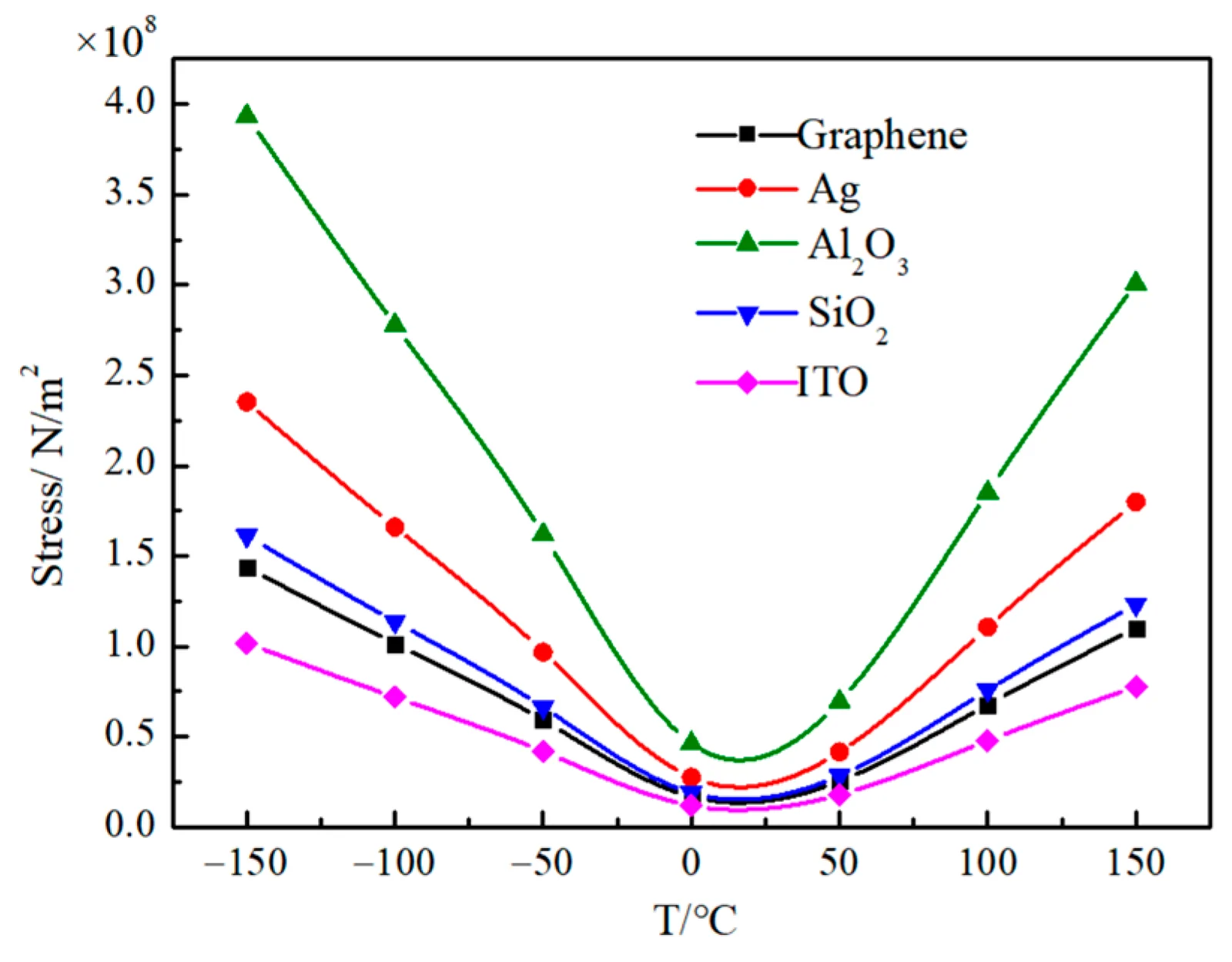
Variation of stress in each functional layer film with temperature.

Figure 5 illustrates the variation of stress in each functional layer of the film at different ambient temperatures. Thermal stress is primarily concentrated in the Al_2_O_3_ layer, with peak stress values of 393 MPa at −150 °C. The high stress in the Al_2_O_3_ layer is mainly attributed to its relatively high elastic modulus and the more significant thermal expansion mismatch with adjacent layers, which induces severe stress concentration under the constraint of extreme low-temperature shrinkage. These results indicate that the Al_2_O_3_ layer are the most vulnerable components of this multilayer film structure under low-temperature space environments. To enhance structural reliability, measures such as modulus gradient design or film thickness optimization should be implemented to alleviate stress concentration.

#### 3.2.2. Stress Distribution Under Laser Irradiation

**Figure 6:**
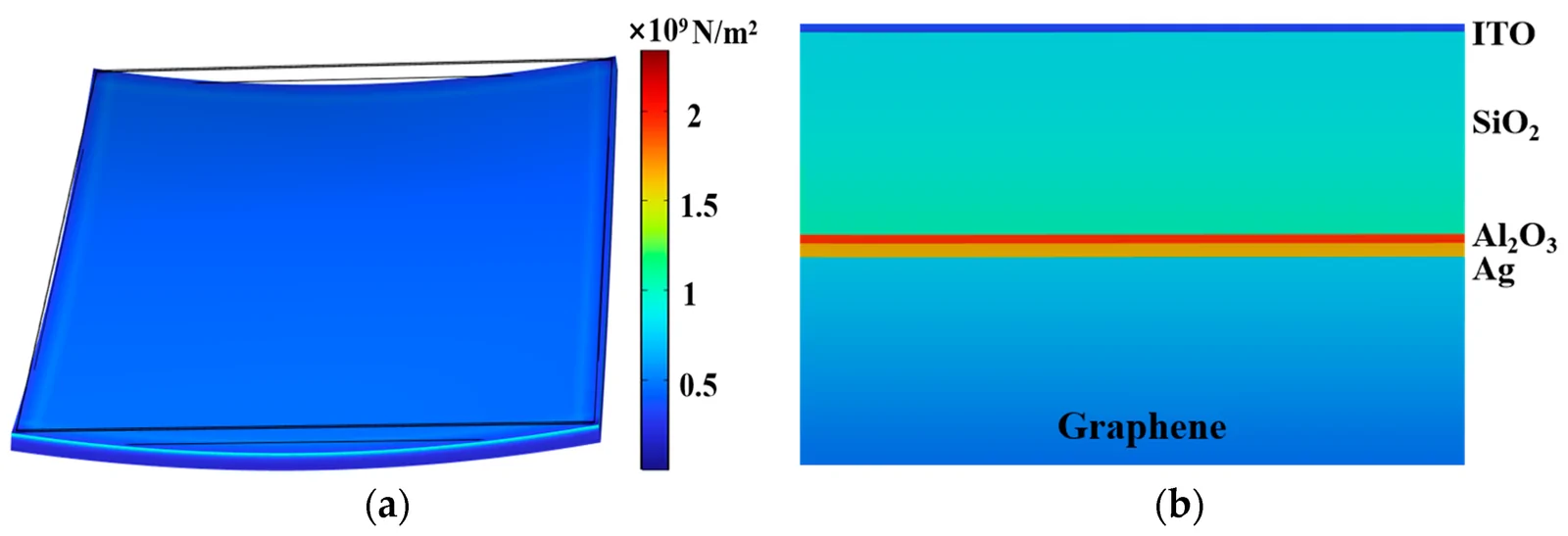
Stress distribution of the film structure under the condition of P = 100 W/cm^2^. (**a**) Overall structural stress distribution of the film. (**b**) Stress distribution along the cross-sectional direction.

High temperature induced by laser irradiation play a dominant role. Under the combined loading of laser irradiation and space environment temperature variation, the laser-induced thermal load dominates the stress response of the multilayer film. While the space environment alone can generate significant thermal stress, the incremental contribution from space temperature fluctuations becomes relatively small when the film is subjected to intense laser heating. Figure 6 shows the stress distribution of the film structure under a laser power density 100 W/cm^2^. The stress under laser irradiation is substantially higher than that without laser loading, and the Al_2_O_3_ layer exhibits the maximum stress.

**Figure 7:**
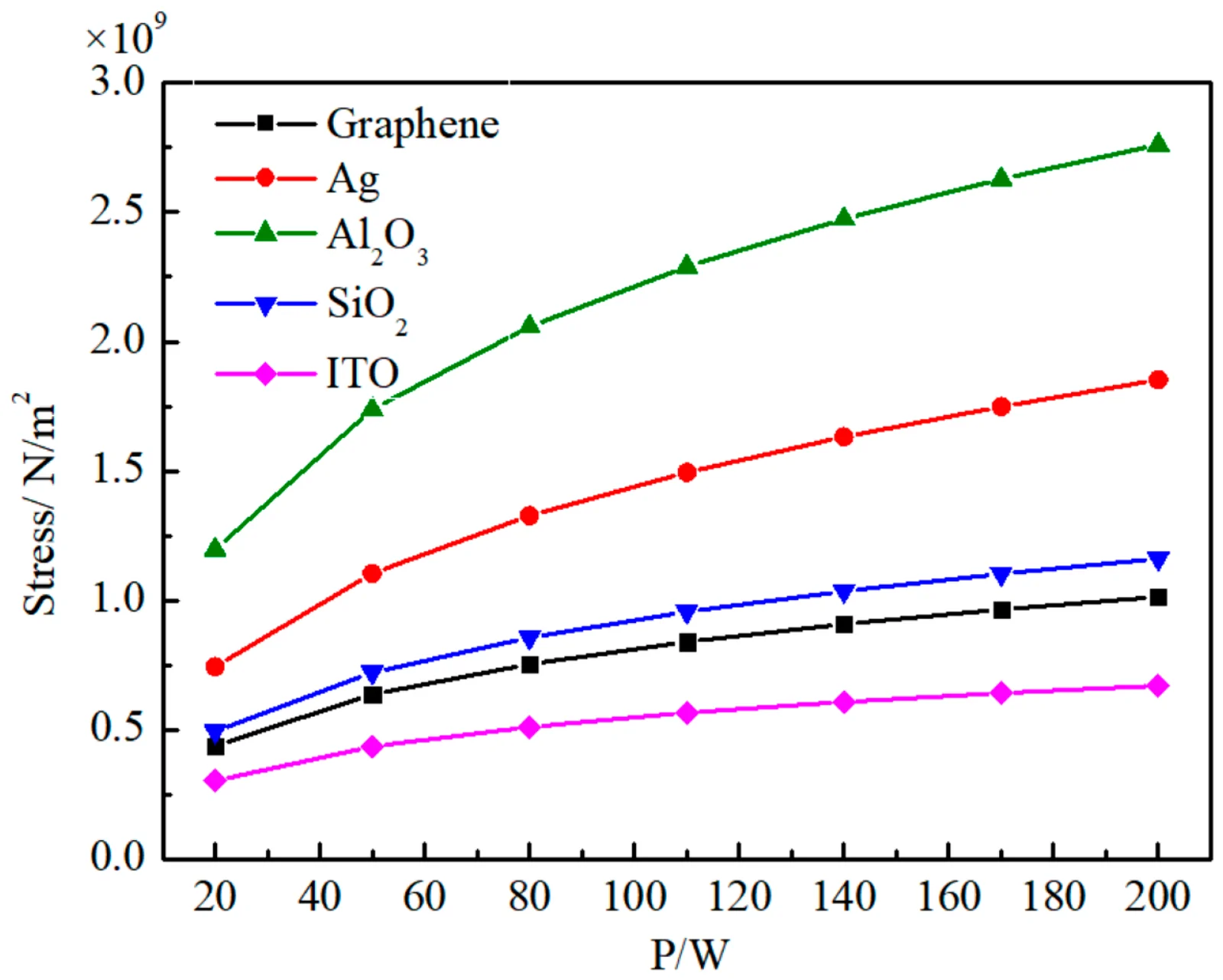
Variation of stress in each functional layer with laser loading power.

Figure 7 shows the variation of stress in each functional layer with laser loading power. The stress of each layer increases nonlinearly with the rise of laser power, and there are remarkable differences in the response amplitude among different layers. Increasing laser power leads to higher energy absorption by the films and a nonlinear growth in peak temperature, which intensifies interlayer temperature gradients. Since thermal stress is proportional to temperature variation Δ*T*, the magnitude of thermal stress inside each layer rises and the interlayer stress gradient increases accordingly. Once the peak stress approaches or exceeds the yield strength or tensile strength of the material, initial damages such as plastic deformation, microcracks and interfacial delamination will initiate. Consequently, elevated laser power causes material property degradation and aggravates film failure by raising the peak temperature, enlarging temperature gradients and increasing stress magnitudes within the multilayer structure. In engineering design, gradient transition layers or other measures to restrain temperature gradients should be adopted for sensitive interfaces to control the risk of film layer failure.

### 3.3. Laser Protection Performance Test Analysis

**Figure 8:**
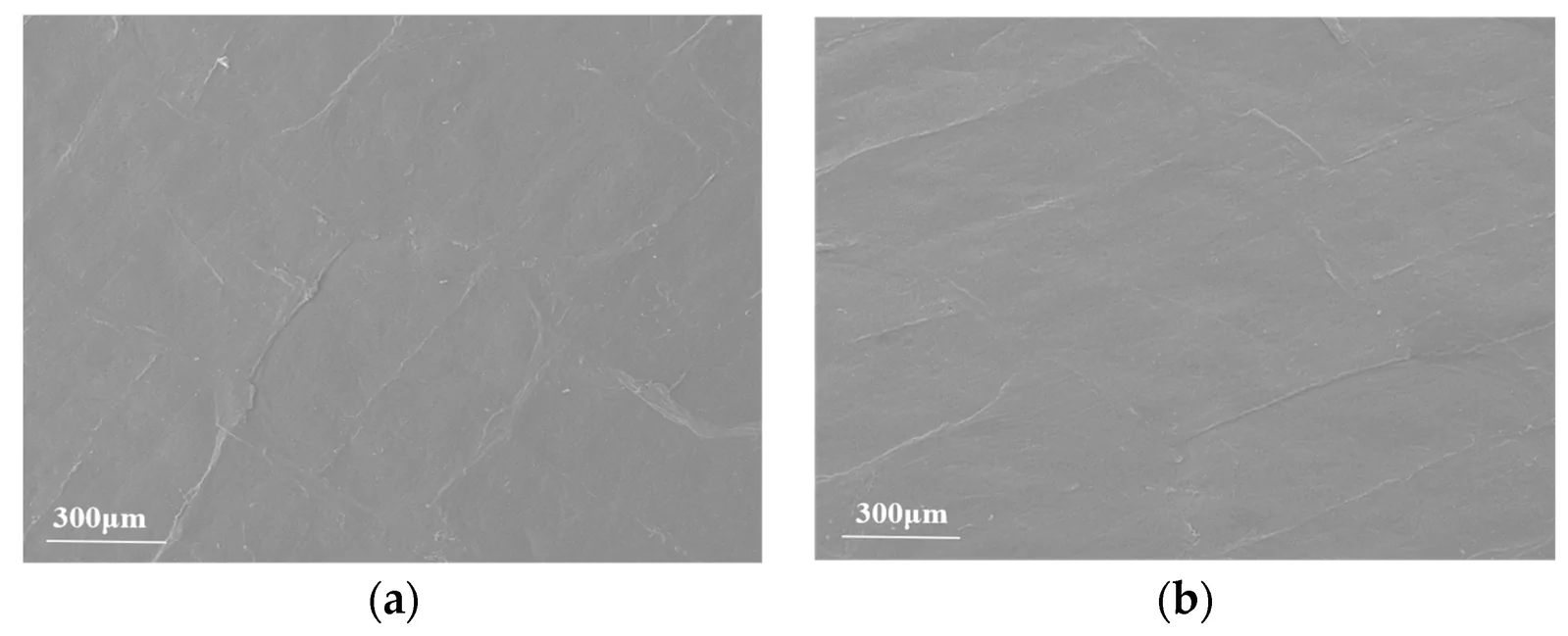
Micromorphology of film surface before and after test (**a**) Before (**b**) After.

To verify that the multilayer-film structure of Graphene/Ag/Al_2_O_3_/SiO_2_/ITO multilayer thin film will not suffer macroscopic failures such as delamination and ablation under space extreme-temperature environments and laser irradiation with a power density of 200 W/cm^2^, laser protection performance tests were conducted under high-low temperature environments ranging from −150 °C to +150 °C. The samples were irradiated with a 200 W/cm^2^ continuous-wave laser (1.064 μm) for 30 s at 150 °C. The microscopic morphologies before and after the test are shown in Figure 8. No macroscopic cracks, wrinkles, blisters, or interfacial delamination were observed on the film surface. Measurements showed that the infrared emittance of the film before and after the test was 0.53 and 0.54, respectively, and the solar absorptance remained at 0.06 in both cases, indicating that the thermal control performance of the film did not undergo significant degradation due to laser irradiation. These results are attributed to the multiple protective mechanisms of the film structure. The Ag layer exhibits an extremely high reflectivity (>95%) at 1.064 μm, reflecting most of the incident laser energy back into space. Meanwhile, Ag has a very high thermal conductivity (approximately 429 W/(m·K)), enabling rapid lateral diffusion of absorbed local heat beyond the spot area, effectively reducing the peak temperature at the spot centre. The Al_2_O_3_ and SiO_2_ layers serve as optical matching layers and thermal barriers, further attenuating the transmitted energy and protecting the substrate material. Under the combined effects of space high-low temperature environments and high-power continuous laser irradiation, the multilayer film structure does not undergo interfacial delamination, penetrating damage, or thermal control performance degradation, thus verifying its engineering feasibility as an integrated laser protection and thermal control material for space applications.

The experimental validation presented in this study is limited to macroscopic observations and bulk thermal property measurements. While the absence of macroscopic failure confirms the structural integrity of the film under the tested conditions, the experimental results do not directly validate the calculated temperature and stress distributions within each individual layer.

### 3.4. Discussion

The present model adopts a continuum-based finite element approach, which treats each layer as a homogeneous medium with averaged material properties. This approach is justified for predicting macroscopic temperature and stress distributions at the device level (film thickness in the sub-micron to micron range, laser spot diameter of 1.2 cm), where the characteristic length scale of the thermal and mechanical phenomena is much larger than the atomic spacing. However, we fully acknowledge that this continuum description cannot capture nanoscale effects such as grain boundary diffusion, dislocation-mediated plasticity, or interface roughness-induced stress concentrations.

The laser irradiation is modeled as a surface heat flux with Gaussian spatial distribution, which assumes that the laser energy is absorbed and converted into heat instantaneously at the surface. This approach does not account for atomistic effects such as phonon-photon coupling, electron-phonon relaxation dynamics, or quantum confinement effects, which may become significant for ultrathin layers or ultrafast pulsed laser irradiation. For the present case of continuous-wave laser irradiation on films with thicknesses of ~100 nm or more, the macroscopic thermal diffusion time is much longer than the electron-phonon relaxation time, justifying the continuum heat source approximation.

The present model assumes a perfect, defect-free multilayer structure with sharp interfaces and no phase change. It does not capture phenomena such as melting, solid-state phase transformation, atomic diffusion across interfaces, segregation of impurity elements, or generation of vacancies and dislocations during laser irradiation. These atomistic and microstructural effects are beyond the scope of a continuum-level FEM model. However, we recognize that under repeated thermal cycling or prolonged laser exposure, such effects could accumulate and ultimately degrade film performance. This is particularly relevant for the Ag/A_2_O_3_ interface, where interdiffusion and void formation may occur at elevated temperatures.

## 4. Conclusions

In this study, the Graphene/Ag/Al_2_O_3_/SiO_2_/ITO multilayer thin film integrating laser protection and thermal control functions is taken as the research object. Using COMSOL Multiphysics based on the finite element method, the temperature and thermal stress distribution of the film under space high-low temperature environments (−150 °C to +150 °C) and laser irradiation are systematically analysed. The results indicate that the weak link of the multilayer film structure is the Ag/Al_2_O_3_ interface, whose stress concentration is jointly influenced by extreme space temperatures and laser power, constituting a key factor restricting the long-term reliability of the film in space laser protection and thermal control integration applications. The laser irradiation tests under high-low temperature conditions demonstrate that under 200 W/cm^2^ laser irradiation, the maximum temperature and maximum stress reached in the film do not cause failure behaviour in the multilayer structure. The research results can provide theoretical reference and data support for structural optimisation, interface design, and long-term reliability evaluation of laser protection-thermal control integrated multilayer films for space applications. The main conclusions are as follows:

Under laser irradiation, the temperature field of the film exhibits a Gaussian distribution in the planar direction. With increasing laser action time, the temperature of each film layer generally shows a trend of rapid initial rise followed by stabilisation. However, significant differences exist in the heating rates and steady-state temperatures among different layers. The higher the laser power, the faster the heating rate and the higher the steady-state temperature attained.

The space high-low temperature environment (−150 °C to +150 °C) induces a significant thermal stress response in the film. The thermal stress distribution exhibits obvious interlayer differences, and the stress amplitude increases with the degree of temperature extremity. Thermal stress is mainly concentrated in the Al_2_O_3_ layer with peak stress values reaching 393 MPa under extreme low temperature at −150 °C. Interlayer thermal expansion coefficient mismatch is the primary cause of thermal stress generation.

Laser loading power has a significant effect on film thermal stress. As laser power increases, the thermal stress amplitude of each film layer increases synchronously, and interlayer temperature gradients and stress concentration phenomena are further intensified. The stress increase in the Ag and Al_2_O_3_ layers is the most significant, and the interfacial stress concentration becomes more prominent, which may further induce failure risks such as interfacial delamination and film cracking.

The laser irradiation test results demonstrate that the Graphene/Ag/Al_2_O_3_/SiO_2_/ITO laser protection-thermal control integrated multilayer film does not exhibit failure behaviours such as film peeling, penetration, or ablation under simulated satellite service conditions. The key thermal control performance of the film shows no significant degradation, verifying the reliability of this multilayer film system for spacecraft space protection engineering applications.

## Figures and Tables

**Table 1:** Thickness and materials of each functional layer.

	Functional Layer	Materials	Thickness
1	Substrate	Graphene	20 μm
2	Reflective layer	Ag	150 nm
3	Transition layer	Al_2_O_3_	100 nm
4	Heat dissipation layer	SiO_2_	2 μm
5	Anti-static layer	ITO	50 nm

**Table 2:** Preparation method of each functional layer.

Functional Layer	PVD Method	Sputtering Gas Pressure/Pa	Sputtering Power/W	Ar Gas Flow/sccm	Voltage Bias/V
Ag	DC magnetron Sputtering	0.2	200	20	100
Al_2_O_3_	IBO + pulsed reactive magnetron sputtering	0.2	600	15	/
SiO_2_	IBO + pulsed reactive magnetron sputtering	0.2	800	15	/
ITO	DC magnetron sputtering	0.8	100	80	100

**Table 3:** Preparation parameters of Al_2_O_3_ and SiO_2_ layer.

Functional Layer	Pulse Frequency/kHz	Positive Pulse Amplitude/V	Positive Pulse Width/μs	Ion Source Voltage/V	Ion Source Discharge Current/A	Oxygen Partial Ratio of Ion Source/%
Al_2_O_3_	25	300	1	300	0.85	86
SiO_2_	15	300	1	300	0.8	82

**Table 4:** Material parameters of each film layer.

Materials	Coefficient of Thermal Expansion ppm/℃	Young’s Modulus GPa	Density kg/m^3^	Poisson’s Ratio	Specific Heat Capacity at Constant Pressure J/(kg·K)	Thermal Conductivity W/(m·K)
Graphene	−6	1000	2260	0.2	715	2000(1n-plane)/3(cross-plane)
Ag	18.9	83	10,500	0.37	235	429
Al_2_O_3_	6.5	400	3965	0.22	730	35
SiO_2_	0.5	70	2200	0.17	730	1.4
ITO	7.2	116	7120	0.23	500	3

**Table 5:** Laser protection performance test parameters.

Test Item	Parameters
Laser type	1.064 μm high-power continuous laser
Spot diameter	1.2 cm
Test vacuum degree	≤5 × 10^−3^ Pa
Temperature	−150 °C, 25 °C, 150 °C
Maximum laser loading power density	≥200 W/cm^2^
Loading duration	30 s

## Data Availability

The original contributions presented in this study are included in the article. Further inquiries can be directed to the corresponding author.
